# Acid ceramidase deficiency: Farber disease and SMA-PME

**DOI:** 10.1186/s13023-018-0845-z

**Published:** 2018-07-20

**Authors:** Fabian P. S. Yu, Samuel Amintas, Thierry Levade, Jeffrey A. Medin

**Affiliations:** 1 Institute of Medical Science, University of Toronto, Toronto, ON Canada; 2 Laboratoire de Biochimie Métabolique, Institut Fédératif de Biologie, CHU Purpan, Toulouse, France; 3 INSERM UMR1037 CRCT, Université de Toulouse, Toulouse, France; 4 Departments of Pediatrics and Biochemistry, Medical College of Wisconsin, Milwaukee, WI USA

**Keywords:** Ceramide, Lysosomal storage disorder, SMA-PME, Sphingolipid, Lysosome, Neuromuscular disease, Sphingolipidosis, Metabolic disorder, Lipid storage, Spinal muscular atrophy, Lipogranulomatosis

## Abstract

Acid ceramidase (ACDase) deficiency is a spectrum of disorders that includes a rare lysosomal storage disorder called Farber disease (FD) and a rare epileptic disorder called spinal muscular atrophy with progressive myoclonic epilepsy (SMA-PME). Both disorders are caused by mutations in the *ASAH1* gene that encodes the lysosomal hydrolase that breaks down the bioactive lipid ceramide. To date, there have been fewer than 200 reported cases of FD and SMA-PME in the literature. Typical textbook manifestations of classical FD include the formation of subcutaneous nodules, accumulation of joint contractures, and development of a hoarse voice. In reality, however, the clinical presentation is much broader. Patients may develop severe pathologies leading to death in infancy or may develop attenuated forms of the disorder wherein they are often misdiagnosed or not diagnosed until adulthood. A clinical variability also exists for SMA-PME, in which patients develop progressive muscle weakness and seizures. Currently, there is no known cure for FD or for SMA-PME. The main treatment is symptom management. In rare cases, treatment may include surgery or hematopoietic stem cell transplantation. Research using disease models has provided insights into the pathology as well as the role of ACDase in the development of these conditions. Recent studies have highlighted possible biomarkers for an effective diagnosis of ACDase deficiency. Ongoing work is being conducted to evaluate the use of recombinant human ACDase (rhACDase) for the treatment of FD. Finally, gene therapy strategies for the treatment of ACDase deficiency are actively being pursued. This review highlights the broad clinical definition and outlines key studies that have improved our understanding of inherited ACDase deficiency-related conditions.

**Electronic supplementary material:**

The online version of this article (10.1186/s13023-018-0845-z) contains supplementary material, which is available to authorized users.

## Background

Dr. Sidney Farber described the first case of “*disseminated lipogranulomatosis”* in a 14-month-old infant at a Mayo Foundation lecture in 1947. Farber later published a case series of three patients in 1952, as a transaction for the 62nd annual meeting of the American Pediatric Society. He later expanded the descriptions in 1957 [1, 2]. Farber originally hypothesized that the disease shared the lipid storage aspects of Niemann-Pick disease as well as the inflammation observed in Hand-Schüller-Christian disease. Although Farber demonstrated an increase in lipids in his early biochemical studies, the main lipid that accumulates in Farber disease (FD), i.e., ceramide, was not identified until 1967, when it was isolated from a biopsy of a patient’s kidney [3]. Acid ceramidase (ACDase), which was first purified in 1963, catalyzes the synthesis and degradation of ceramide into sphingosine and fatty acid [4]. In 1972, Sugita and colleagues established that ACDase activity was not detectable in post-mortem tissue from a FD patient [5]. In 1996, the *ASAH1* gene that encodes ACDase was fully sequenced and characterized [6].

Our literature search spans 70 years and identifies 201 patients described as having ACDase deficiency (Tables 1 and 2). We included cases that were published in English, French, German, Chinese, Russian, and Arabic. While most of the cases we reviewed involved the classical FD phenotype, some were related to the rare motor neuron disease, SMA-PME (Tables 1 and 2). In this review, we will outline the clinical spectrum of ACDase deficiency and summarize key biochemical, genetic, and clinical studies related to this disorder.

**Table 1:** Cases Analyzed

	Cases mentioned in literature	Average age of onset	Average age of last documentation	Average age of death
Classic & Severe FD	102	5.8 ± 4.6 M (70)	1.9 ± 1.8 Y (18)	2.6 ± 6.0 Y (61)
Mild & Intermediate FD	40	1.5 ± 1.4 Y (34)	14.9 ± 18.1 Y (26)	14.3 ± 8.1 Y (8)
FD (Unspecified)	16	–	–	–
SMA-PME	23	5.8 ± 4.2 Y (18)	16.5 ± 5.7 Y (15)	14.4 ± 3.0 Y (5)
SMA-PME Like	20	8.9 ± 7.38 (20)	22.68 ± 17.8 Y (10)	21.9 ± 17.2 Y (10)
Total FD	158			
Total SMA-PME	43			

Total number of cases of ACDase deficiency reported from 1952 to 2018 by clinical presentation, severity, and average ages. Unspecified represents cases in which a diagnosis was made but insufficient clinical information was provided for placement in a clinical category. (See Additional file 1 for methodology). *M* months, *Y* years, number in brackets indicates the total number of cases included to calculate the average age and standard deviation

**Table 2:** Main Clinical Features Present in Cases Related to ACDase Deficiency

Variants	Cases with clinical details	Nodules	Joint contractures	Hoarse voice	Hepatosplenomegaly	Neurological and behavioral	Respiratory	Motor neuron/Muscle weakness	Ocular	Bone	Myoclonus & seizures
Classic and Severe FD	79	95%	96%	90%	38%	62%	38%	32%	22%	18%	19%
Mild & Intermediate FD	36	94%	97%	72%	3%	22%	25%	25%	8%	31%	14%
SMA-PME	20	0%	0%	0%	0%	60%	45%	100%	0%	0%	100%
SMA-PME (like)	19	0%	0%	0%	0%	32%	26%	95%	0%	0%	100%

Percentage representations of common clinical features in the literature for FD and the SMA-PME variant of ACDase deficiency

### Traditional classifications of Farber disease

**Fig. 1:**
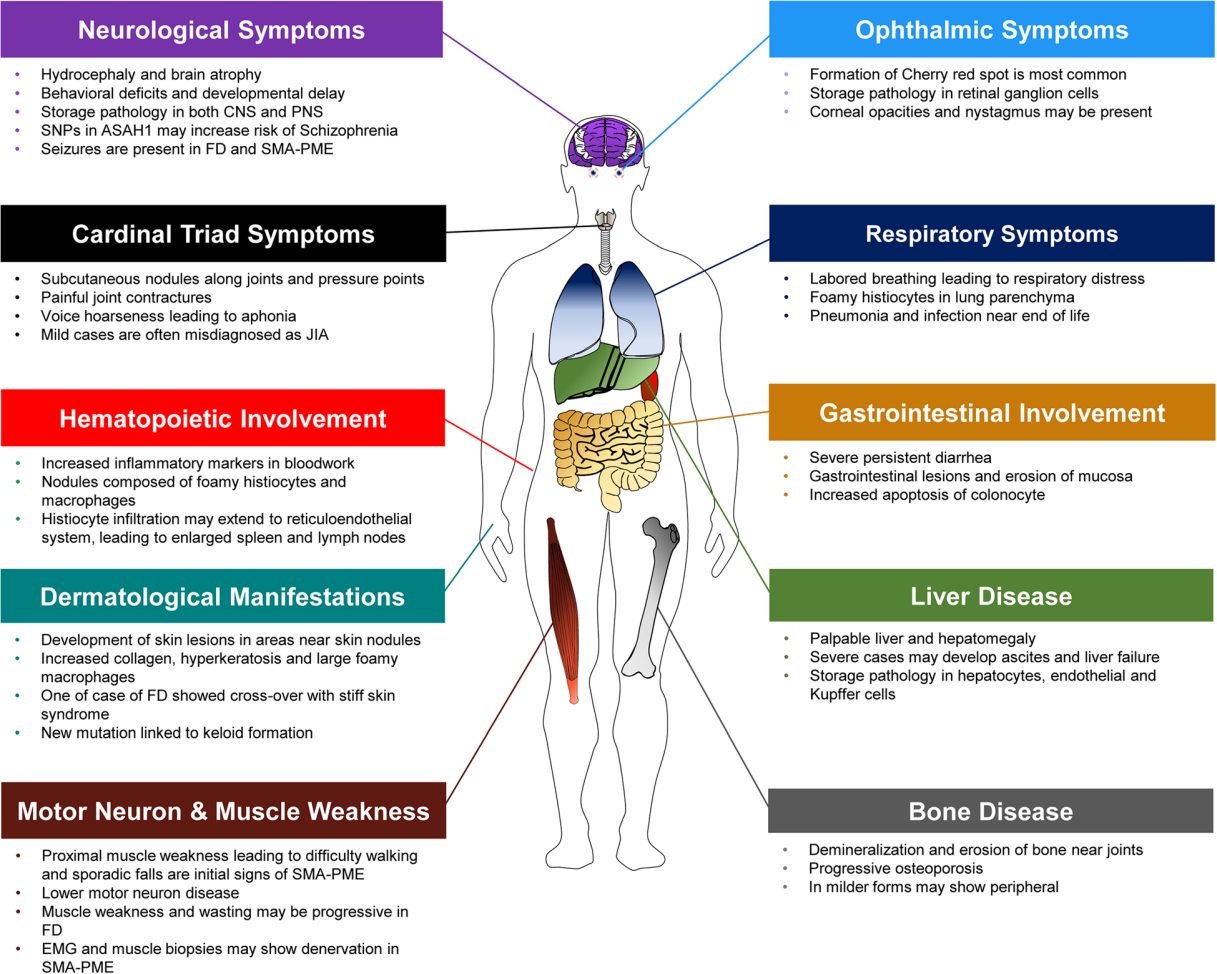
The typical clinical manifestations by organ type that have been reported in cases of Farber Disease (FD) and SMA-PME in the published literature. FD symptoms organized by neurological symptoms, ophthalmic symptoms, cardinal triad symptoms, respiratory symptoms, hematopoietic symptoms, gastrointestinal involvement, dermatological manifestations, liver disease, motor neuron and muscle weakness, and bone disease phenotypes

Farber disease (FD; OMIM #228000), also known as Farber’s lipogranulomatosis, is an ultra-rare lysosomal storage disorder (LSD). It is caused by mutations in *ASAH1*, which lead to decreased ACDase activity and in turn, to ceramide accumulation and various pathological manifestations (Fig. 1). Moser and colleagues first categorized FD into 5 subtypes in a review in 1989, later adding two other phenotypes [7, 8]. Type 1, also termed the “classical” variant of FD, includes patients with the cardinal symptoms of subcutaneous nodules, joint contractures, and voice hoarseness. These patients may also develop enlarged liver and spleen along with neurological and respiratory complications [8, 9]. Traditionally, Type 1 FD patients exhibit symptoms during infancy and typically do not live past the age of 2–3 years [2, 10]. Types 2 and 3 FD patients have been termed the “intermediate” and “mild” variants, respectively; patients with these phenotypes usually have a longer lifespan due to reduced neurological involvement. However, Types 2 and 3 FD patients suffer from subcutaneous nodules, joint contractures, and aphonia due to inflammation. Types 4 and 5 FD patients have severe disease manifestations. Type 4 is associated with the “Neonatal-Visceral” variant, wherein neonates experience severe organomegaly and visceral histiocytosis [8, 11]. Type 5 is the “Neurological Progressive” variant, which is manifested by progressive neurological deterioration and seizures. Nodules and joint involvement are present in Type 5; however, they are less severe. Type 6 FD is termed “Combined Farber and Sandhoff Disease variant.” In this single co-incidental case, the patient had combined Farber and Sandhoff (OMIM #268800) diseases [12]. The patient presented with clinical signs of FD, and demonstrated a deficiency in both ACDase and hexosaminidases A and B [12]. Finally, Type 7 FD is termed “Prosaposin Deficiency.” This phenotype was identified in one patient and his infant sibling [13]; a mutation was identified in the precursor protein of saposins (i.e., prosaposin, encoded by the *PSAP* gene) [14]. A total of 4 saposins have been identified, and these proteins, along with the GM2 ganglioside activator protein, collectively belong to a group of sphingolipid activator proteins (SAPs). Only a handful of patients with Type 7 FD have been reported [15]. Similar to Type 6 FD, these patients often have multiple enzyme deficiencies, such as reduced glucocerebrosidase, galactocerebrosidase and ceramidase activities. While patients with prosaposin deficiency may show some biochemical and clinical signs that overlap with FD, it is considered a separate disease (OMIM #176801). Increasingly, many of the more recently reported cases simply identify FD as either the classic childhood or the mild and attenuated form [16–18]. Since some of these subtypes are rare and represent separate conditions, an updated classification should be considered to incorporate the existing and emerging phenotypes of ACDase deficiency.

## Biochemistry, genetics and diagnosis

### Acid ceramidase and ceramides

**Fig. 2:**
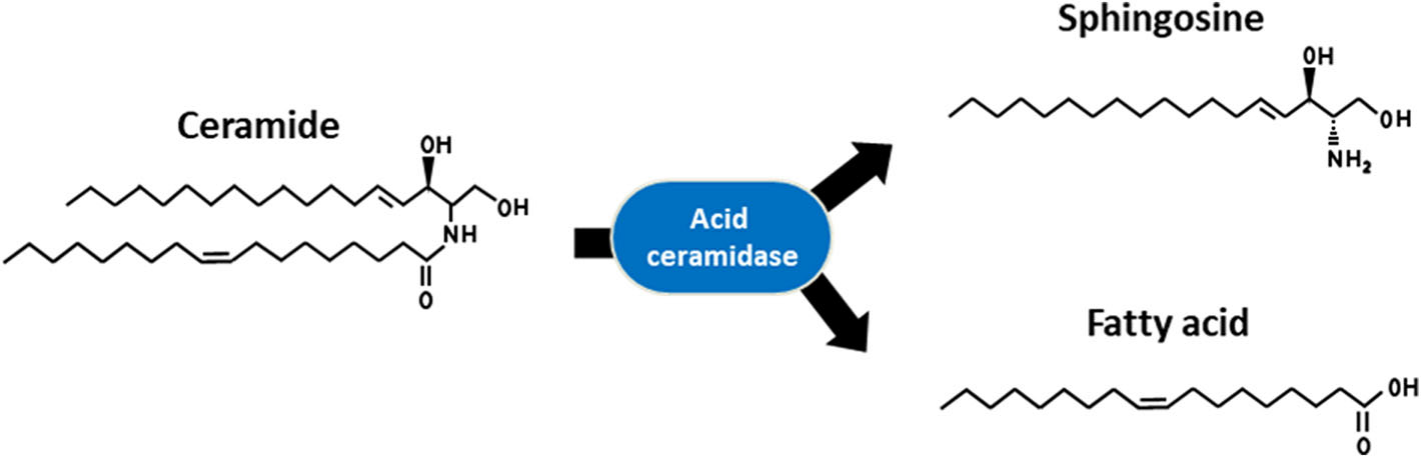
Reaction schema of the hydrolysis of ceramide by acid ceramidase into sphingosine and free fatty acid

Acid ceramidase (ACDase) (E.C. #3.5.1.23) was first identified in 1963 by Gatt in rat brain extracts, where he demonstrated that ACDase was the catalyst for the hydrolysis of the amide bond of ceramides (Fig. 2) [4]. The optimal pH of ACDase is 4.5–5, and this enzyme is responsible for the hydrolysis of ceramide into a sphingosine and a free fatty acid. Due to the low pH, it had been suggested that the enzyme may have a role in the lysosomal system [19]. The first large purification of the enzyme was not performed until 1995 using human urine samples [20]. The purified enzyme was later identified as a heterodimer consisting of an α (13 kDa) and a β (40 kDa) subunits. Studies utilizing the first anti-ACDase polyclonal antibody revealed that ACDase is initially synthesized as a precursor polypeptide and then post-transcriptionally modified and processed into the α and β subunits within the lysosome [21]. These studies also revealed that cleavage into its subunits is essential for enzymatic activity. Later studies using rhACDase showed that cleavage of the precursor polypeptide occurs through an autoproteolytic reaction that is dependent on the cysteine residue 143 [22, 23]. Recently the crystal structure of mammalian ACDase was elucidated for both the proenzyme and the mature form [24]. This study showed that autocleavage of ACDase triggers a conformational change that uncovers the active site for ceramide entry [24]. Additional modeling demonstrated distinct catalytic mechanisms for autocleavage and for hydrolysis of substrate [24]. ACDase, like other enzymes, also exhibits a reverse reaction, in which ACDase can use C12:0 fatty acid and sphingosine to form ceramide at a pH of 6 rather than the lower pH of 4.5 [25]. Similar to other acidic hydrolases, ACDase is tagged with a mannose-6-phosphate residue for transport to the lysosomal compartment.

Ceramide lies in one of the central steps in the breakdown and formation of other sphingolipids [26, 27]. Many of these lipids, such as sphingomyelin and complex glycolipids, play important roles in cell membranes [26, 27]. Other sphingolipids, such as glucosylceramides and sulfatides, are essential for the formation and breakdown of myelin [28]. Many species of ceramides exist, and each species is defined by the length, saturation, and hydroxylation of both the fatty acid and sphingoid base moieties [26]. Due to the central role of ceramide in sphingolipid metabolism, any imbalance in ceramide metabolism may lead to significant downstream effects and disease. This topic has been covered in a number of reviews [29–33].

### Prevalence of ACDase deficiency

FD is an ultra-rare disease where the prevalence and incidence are not accurately known. According to the epidemiological Orphanet report, FD (ORPHA 333) has a predicted prevalence of < 1/1,000,000 (https://www.orpha.net/consor/cgi-bin/OC_Exp.php?Expert=333). While no formal comprehensive international epidemiological study has been performed for FD, one recent quantitative analysis of 96 case studies found that India and the USA had more than 10 reported cases, followed by Saudi Arabia, Germany, France, and Italy all of which had between 6 to 10 cases [34].

Based on our literature search, we identified 158 reported cases of FD between 1952 and 2018 (Table 1). SMA-PME currently has its own OMIM entry and is usually categorized as a subtype of SMA [35]. We identified 23 cases of SMA-PME associated with mutations in *ASAH1* gene since Zhou and colleagues first reported this finding [36]. Due to the rare nature of both disorders, they originally appeared to be two very separate conditions; however, as more cases of each are characterized, the clinical pictures are starting to overlap. For example, a recent case described a patient who presented with muscle weakness typical of SMA-PME, but who also had joint pain synonymous with FD [37]. Additionally, several cases of FD have shown neurological involvement such as delayed mental development, seizures, and muscle weakness as predominant pathologies [9, 38–40].

### Genetics and mutations

**Fig. 3:**
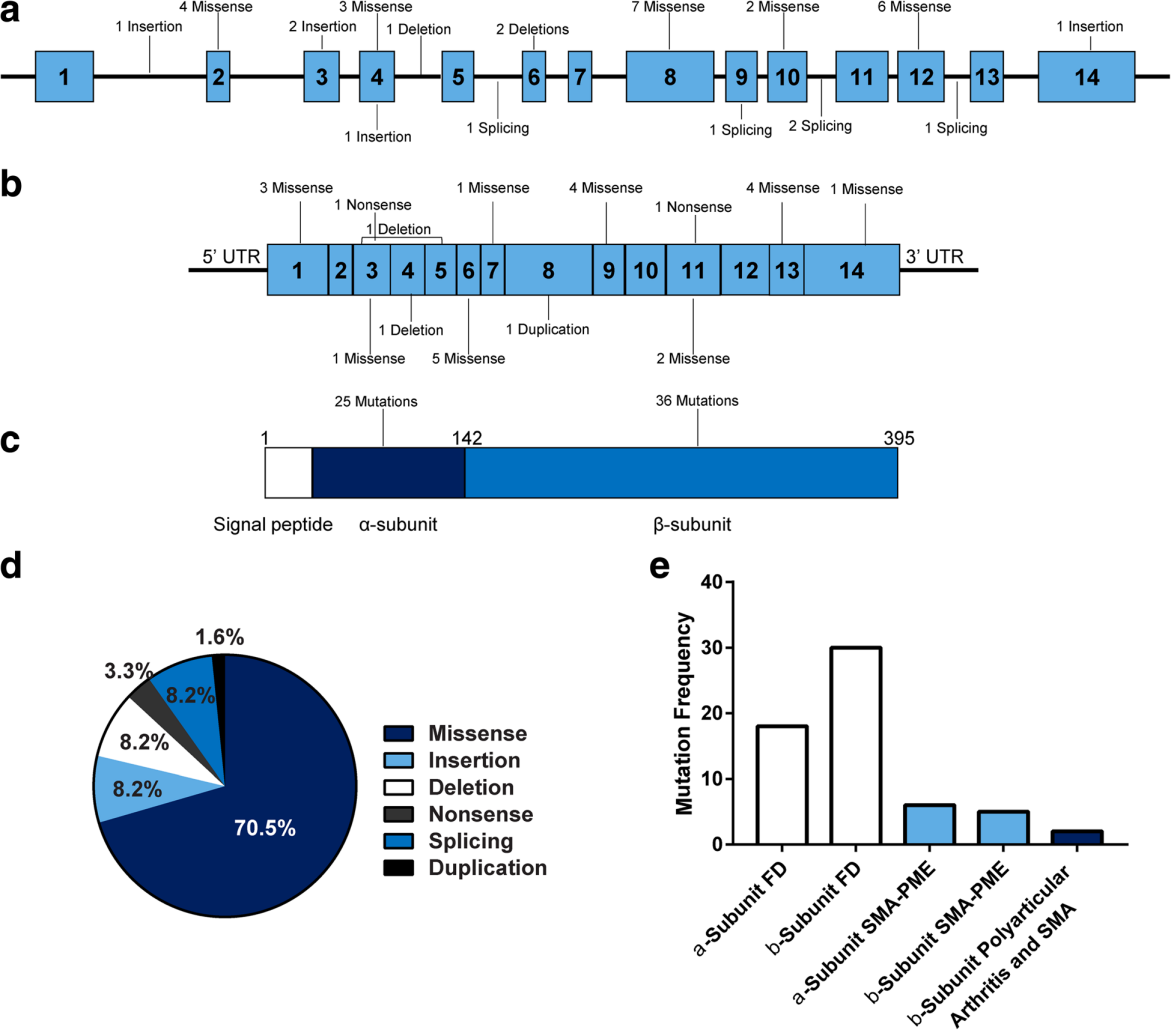
Structure of the human *ASAH1* gene with the protein and distribution of mutations. **a** *ASAH1* genomic structure. **b** *ASAH1* mature transcript structure. **c** Schematic of the ACDase protein with annotations for the signal peptide, α-subunit, and β-subunit. **d** Percentages of the reported 65 *ASAH1* mutations by type for FD and SMA-PME. **e** Frequency of mutations by subunit and reported disease phenotype

The human acid ceramidase gene (*ASAH1*) is approximately 30 kb in total length. It contains 14 exons that range from 46 to 1200 bp long and is mapped to the short arm of chromosome 8 (8p21.3/22) [41]. The first mutation identified, c.665C > A (p.T222K), was from a patient with a severe form of FD [6]. Based on the literature, we identified 61 pathologic mutations leading to FD or SMA-PME. These mutations are summarized in Tables 3 and 4. Additionally, at the time this review was being written, more than 120 genetic variants had been submitted to the NCBI ClinVar public archive [42]. While a number of these represent published mutations with a pathogenic role, most of the variants were submitted by clinical diagnostic testing facilities and did not include confirmed pathological details. Therefore, our curated list is likely an under-representation of all the sequenced pathologic mutations in FD. Nonetheless, several observations can be extrapolated. Mutations have been identified throughout the *ASAH1* gene, but most of the mutations appear to be missense mutations (Fig. 3a-e). Among the recorded mutations that result in the diagnosis of FD, a majority is located within the β-subunit. Eighteen patients were identified to have a mutation in exon 8 and 9 patients had mutations in exon 13. In contrast, a larger number of mutations in SMA-PME have been identified within the α-subunit. One interesting observation is that the T42A and T42M mutations in exon 2 accounted for more than half of the total number of reported cases of SMA-PME. While some of these cases are siblings, they have also occurred within independent families [36, 43–47]. There is currently no definitive genotype-to-phenotype relationship in the noted mutations, which is especially true based on the observation that one patient with SMA-PME and another with FD had the same Y137C mutation [48, 49]. Another patient presented with polyarticular arthritic symptoms synonymous with FD and later developed muscle weakness with no PME [37]. These examples indicate that mutations in *ASAH1* can result in a broad range of phenotypes.

**Table 3:** Reported Mutations in *ASAH1* that result in FD

DNA Change	Mutation type	Locus	Amino acid change	Allelic status	ACDase activity	Number of cases	Reference
c.66G > C	Missense	Exon 1	p.Q22H	NI	NI	1	[195]
c.67C > G	Missense	Exon 1	p.H23D	NI	NI	1	[195]
c.92G > T	Missense	Exon 2	p.C31F	Homoallelic	NI	2	[49, 115]
c.107A > G	Missense	Exon 2	p.Y36C	Homoallelic & Heteroallelic	NI	4	[49, 196]
c.126-3941_382 + 1358del	Deletion	Exon 3–5	p.Y42Rfs*10	Heteroallelic	undetectable	1	[197]
c.174_175InsC	Insertion	Exon 3	p. E64*	Heteroallelic	NI	1	[169]
c.212C > A	Missense	Exon 3	p.P71Q	Heteroallelic	NI	1	[198]
c.256_257insA	Insertion	Exon 4	p.T86Nfs*13	Heteroallelic	NI	1	[17]
c.290_292delTGG	Deletion	Exon 4	p.V96del	Homoallelic	37%	1	[199]
c.290 T > A	Missense	Exon 4	p.V97E	Heteroallelic	35%	1	[199]
c.290 T > G	Missense	Exon 4	p.V97G	Homoallelic	NI	2	[120]
c.314 T > C	Missense	Exon 4	p.L105P	Heteroallelic	NI	1	[17]
c.383-16_383-12delTTTTC	Deletion	Intron 5	–	Heteroallelic	NI	1	[131]
c.372 T > A	Missense	Exon 6	p.D124E	Heteroallelic	NI	1	[198]
c.408 T > A	Missense	Exon 6	p.F136L	Heteroallelic	NI	1	[131]
c.412G > T	Deletion	Exon 6	p.E139*	Heteroallelic	NI	1	[169, 196]
c.410A > G	Missense	Exon 6	p.Y137C	Homoallelic	NI	1	[49]
c.410_411delAT	Deletion	Exon 6	p.Y137*	Heteroallelic	NI	2	[114, 169]
c.413A > T	Missense	Exon 6	p.E138V	Homoallelic & Heteroallelic	< 5%	5	[41, 50, 169, 196]
c.457 + 4A > G	Splicing	Intron 6	–	Homoallelic	NI	2	[121, 131]
c.502G > T	Missense	Exon 7	p.G168W	Homoallelic	undetectable	1	[126]
c.505 T > C	Missense	Exon 8	p.W169R	Homoallelic & Heteroallelic	< 10%	7	[49, 53, 93, 131]
c.538G > A	Missense	Exon 8	p.E180K	Heteroallelic	NI	1	[131]
c.544C > G	Missense	Exon 8	p.L182V	Homoallelic	NI	4	[107, 131]
c.593 T > C	Missense	Exon 8	p.V198A	Heteroallelic	NI	1	[131]
c.626G > A	Missense	Exon 8	p.G209D	Heteroallelic	NI	1	[169]
c.665C > A	Missense	Exon 9	p.T222K	Homoallelic	< 5%	1	[6, 196]
c.677G > C	Missense	Exon 9	p.R226P	Heteroallelic	NI	1	[131]
c.703G > C	Missense	Exon 9	p.G235A	Homoallelic & Heteroallelic	2%	3	[131, 199]
c.704G > A	Missense	Exon 9	p.G235D	Heteroallelic	NI	1	[114]
c.704-2A > G	Splicing	Exon 9	–	Homoallelic	NI	1	[49]
c.760A > G	Missense	Exon 10	p.R254G	Homoallelic & Heteroallelic	< 10%	4	[41, 54, 93, 169, 198]
c.770 T > C	Missense	Exon 10	p.L257P	Homoallelic	NI	1	[55]
c.833C > T	Missense	Exon 11	p.P278L	Homoallelic	NI	2	[8, 169]
c.917 + 4A > G	Splicing	Intron 11	–	Heteroallelic	NI	1	[197]
c.917 + 5G > A	Splicing	Intron 11	–	Homoallelic	NI	1	[169]
c.958A > G	Missense	Exon 12	p.N320D	Homoallelic	< 15%	1	[196]
c.959A > G	Missense	Exon 12	p.N320S	Homoallelic	NI	1	[131]
c.991G > A	Missense	Exon 12	p.D331N	Heteroallelic	NI	1	[169, 196]
c.997C > T	Missense	Exon 12	p.P333C	Homoallelic & Heteroallelic	NI	3	[49, 92]
c.997C > G	Missense	Exon 12	p.P333G	Heteroallelic	NI	4	[49, 131]
c.998G > A	Missense	Exon 12	p.P333H	Homoallelic	NI	1	[131]
c.1085C > G	Missense	Exon 13	p.P362R	Homoallelic	< 5%	2	[41]
c.1084C > A	Missense	Exon 13	p.P362T	Heteroallelic	NI	1	[131]
c.1096A > C	Missense	Exon 13	p.K366Q	Heteroallelic	NI	2	[49, 53]
c.1105G > A	Missense	Exon 13	p.V369I	Heteroallelic	NI	1	[199]
c.1098 + 1G > T	Splicing	Intron 13	p.N348_K366del	Heteroallelic	NI	1	[196]
c.1175A > G	Missense	Exon 14	p.R254G	Heteroallelic	NI	1	[169]
c.1186_1187insT	Insertion	Exon 14	p.*396L	NI	NI	1	[195]

List of *ASAH1* mutations reported in the literature that result in FD. Only pathogenic mutations are included. The number of cases column indicates any case in which one allele carries a mutation. Patients with compound mutations that are pathogenic are listed twice. The listed residual enzyme activity is expressed as a percent of the normal control; *NI* not indicated

**Table 4:** Reported Mutations in *ASAH1* that result in SMA-PME

DNA Change	Mutation type	Locus	Amino acid change	Allelic status	ACDase activity	Number of cases	Reference
c.77C > G	Missense	Exon 1	p.P26R	Heteroallelic	NI	1	[200]
c.124A > G	Missense	Exon 2	p.T42A	Homoallelic & Heteroallelic	< 10%	4	[43, 47, 49]
c.125C > T	Missense	Exon 2	p.T42M	Homoallelic	32%	12	[36, 44–46, 153, 201]
*c.125 + 1G > A*	Insertion	Intron 2	–	Heteroallelic	NI	2	[49, 200]
c.177C > G	Nonsense	Exon 3	p.Y59*	Heteroallelic	NI	1	[44]
c.223_224insC	Insertion	Exon 3	pV75Afs*6	Heteroallelic	NI	1	[44]
c.410A > G	Missense	Exon 6	p.Y137C	Heteroallelic	NI	1	[48]
c.456A > C	Missense	Exon 6	p.K152N	Heteroallelic	< 20%	5	[44, 48, 49, 91]
c.518A > T	Missense	Exon 8	p.N173I	Heteroallelic	< 10%	1	[37]
c.536C > T	Missense	Exon 8	p.T179I	Heteroallelic	NI	3	[43, 49]
c.594_599dupCTTCAA	Duplication	Exon 8	F199_K200dup	Heteroallelic	< 10%	1	[37]
c.850G > T	Nonsense	Exon 11	p.G284X	Heteroallelic	< 10%	1	[145]
c.886C > T	Missense	Exon 11	p.R296X	Heteroallelic	< 20%	1	[91]

List of *ASAH1* mutations reported in the literature that result in SMA-PME. Only pathogenic mutations are included. The number of cases column indicates any case in which one allele carries a mutation. Patients with compound mutations that are pathogenic are listed twice. The listed residual enzyme activity is expressed as a percent of the normal control; *NI* not indicated

In the same year that the relationship between *ASAH1* and SMA-PME was established, another report also demonstrated that Han Chinese patients diagnosed with schizophrenia showed a down-regulation of the *ASAH1* gene. Furthermore, this study identified two *ASAH1* SNPs (rs7830490, and rs3753118) associated with schizophrenia [50]. This observation was also reported in a separate and larger study that analyzed the exomes of 12,332 Swedish individuals, of which 4,877 were affected by schizophrenia [51]. That study found patients with schizophrenia had a higher abundance of ultra-rare variants, of which 7 SNPs loci were in the *ASAH1* gene (rs781294134, rs759037498, rs761518207, rs13785393, rs764327759, rs757058563, and rs773025886) [51]. One final example of the broad ACDase deficiency phenotype that can occur is the aforementioned case regarding keloid formation and the L386P mutation in *ASAH1* [52].

### Clinical diagnosis

FD is inherited in an autosomal recessive manner. Due to its rarity, prenatal screening/neonatal testing is typically not performed unless an older sibling has been previously diagnosed. Consideration of FD is typically based on the manifestation of the cardinal triad symptoms: 1) subcutaneous nodules, 2) joint pain, and 3) voice hoarseness [8]. Diagnosis of the mild and attenuated variants of FD is more troublesome since one or more of the featured symptoms may be absent or missed at the time of diagnosis. One report describes a patient who had no apparent subcutaneous nodule formation until the age of 12 years [53]. As mentioned, other cases have been misdiagnosed as juvenile idiopathic arthritis (JIA) [54, 55]. In fact, one cohort study demonstrated that as many as 71% of FD with mild to intermediate variants of FD were initially misdiagnosed as JIA [55]. Thus, the incidence of FD is likely underestimated. JIA patients who have symptoms consistent with the cardinal triad should also be encouraged to be tested for FD as part of their diagnosis.

In addition to JIA, the differential diagnosis includes rheumatoid arthritis, juvenile hyaline fibromatosis, and multi-centric histiocytosis, due to the similarity in joint and subcutaneous manifestations [8]. In severe cases, misdiagnosis may also occur since the main clinical picture is histiocytosis and hepatosplenomegaly [56]. In these cases, the cardinal symptoms are often masked or have not yet developed since these severe symptoms usually manifest early in infancy.

While the diagnosis of FD often requires further biochemical and genetic analyses, several case reports originating from developing countries have relied on clinical and histological diagnoses due to limited resources and lack of access to specialized diagnostic centers. Morphologic characterization is often achieved through analyses of subcutaneous nodules or other biopsied tissue. Common features reported include the presence of granulomas and large lipid-laden macrophages. A variety of studies have used ultrastructural analyses to demonstrate the presence of semi-curvilinear inclusions, also known as ‘Farber bodies, Banana bodies, and Zebra bodies’, in various tissue types [57–60].

### Biochemical and genetic diagnosis

One method that has been adopted to assist in the diagnosis of FD is a lipid loading test on cultured living cells. In this technique, exogenously labeled sphingolipids are added to patient cells and ceramide turnover is assessed. A variety of precursors have been used, including [^14^C] stearic acid–labeled cerebroside sulfate in skin fibroblasts, [^3^H] sphingomyelin in both patient cultured fibroblasts and transformed lymphocytes, and [^14^C] serine, a precursor substrate in the de novo ceramide synthesis pathway, to demonstrate impaired ceramide degradation in FD [61–63].

The most common biochemical method in use for a definitive diagnosis of FD is an enzyme activity assay using cultured patient fibroblasts. Enzyme activity in FD cells is typically < 10% of normal controls, whereas SMA-PME cells have been reported to have as much as 32% of the activity of controls [8, 36]. In addition to fibroblasts, the enzyme activity assay has been tested using leukocytes, plasma, post-mortem tissue, and cultured amniocytes from prenatal testing [64–68]. Conventionally, ACDase activity is determined by the use of either radiolabeled ceramides or fluorescent ceramide analogues. Many of these compounds are not water-soluble and require the use of detergents in addition to specialized technical equipment for analyses [20, 64, 68–73]. This drawback means that diagnosis is available in only a very limited number of laboratories. Currently, ACDase activity can be detected with the use of the fluorogenic substrate Rbm14–12 in a 96-well plate in a high-throughput manner [74, 75].

Quantitation of excess ceramides is another method to assist in the diagnosis. The diacylglycerol kinase assay was commonly used in early studies to measure total ceramides, but it was limited because it did not provide information about individual ceramide species [76]. Later, chromatographic methods such as thin-layer chromatography and high-performance liquid chromatography, were also employed to quantify ceramides [77–79]. The major drawbacks to these methods were the requirement for radiolabeling or fluorophore incorporation. These methods were found to be difficult to perform and provided limited information on individual ceramide species. Mass spectrometry (MS), in particular electrospray ionization mass spectrometry (ESI/MS), is currently the most sensitive method for the discrimination and detection of sphingolipids [80–85]. These methods have been implemented to demonstrate excess ceramide in biopsy samples of subcutaneous nodules, post-mortem liver samples, urine samples, and cultured cells [8, 49, 57, 71, 77, 86–89].

### Genetic testing

The first few mutations in *ASAH1* were identified in patient cultured fibroblasts and required amplification of genomic sequences of *ASAH1* and a combination of PCR and Sanger sequencing [6, 90]. Exome sequencing is now commonly performed and, in conjunction with biochemical assays, provides a conclusive diagnosis of ACDase deficiency [37, 91]. This is particularly informative in patients with non-classical FD, SMA-PME, and in cases in which the symptoms are suggestive of ACDase deficiency but have atypical presentations [47, 48, 92, 93].

### Biomarkers

Increased inflammation and the formation of histiocytes are common in many cases of FD. Recent studies from our laboratories identified monocyte chemoattractant protein 1 (MCP-1) as a potential biomarker [94, 95]. A multiplex cytokine analysis was performed using plasma obtained from FD, JIA, and FD patients who underwent HSCT. This study demonstrated an elevation of MCP-1 in FD samples, but low levels in JIA and normalized levels in FD patients who underwent HSCT [94]. MCP-1 may thus be a beneficial biomarker and could help address the issue of misdiagnosis in mild cases of FD.

Another potential biomarker for the diagnosis of FD is C26:0 ceramide, which was identified by lipid MS quantification of ceramides from lipids extracted from dried blood spots [49]. Two isoforms of C26:0 have been described, with isoform 1 being expressed at a significantly higher level in the newborn (0–6 months) cohort versus the juvenile (0.5–4 years) and adult (> 17 years) cohorts. No details were provided regarding the clinical phenotypes of these patients, but the application of a platform for bloodspot analysis for newborns could be an important step in earlier diagnosis of ACDase deficiency.

## The diverse signs and symptoms in ACDase deficiency

### Cardinal triad symptoms of FD

The classical triad of symptoms that manifest in FD is the formation of subcutaneous nodules, painful and swollen joints, and the development of a hoarse voice and aphonia [9]. Subcutaneous nodules are palpable and may cause hyperesthesia; this is often evident within the first few weeks of nodule development in severe cases [2, 10, 12]. However, nodule formation may present later in life in attenuated forms of the disease [55, 96, 97]. Nodules typically appear on joints and over pressure points. With time, the nodules may thicken and increase in size and number, causing significant swelling. Joint contractures can manifest in a number of locations, ranging from the interphalangeal, metacarpal, wrist, elbow, knee, ankle, and facet joints of the spine [98–101]. Joint contractures are progressive, and the resulting lack of movement can severely limit mobility for some patients [99, 102]. The development of a hoarse voice also occurs as a result of nodule formation in the larynx. Infants are often reported to have a weak cry, which progresses to dysphonia and eventually an inability to speak [98]. The formation of the nodules in the upper airway may also expand to the epiglottis and cause swelling, which results in feeding and respiratory difficulties [10, 89, 103]. If the nodule formation is extreme, tracheostomy may be required [10, 86].

While a definitive diagnosis of FD ideally incorporates the measurement of ACDase enzyme activity, accessibility to the assay and/or a reference diagnostic center is an issue in certain developing countries [100, 104, 105]. In these circumstances, diagnosis of FD is made by relying on the triad symptoms and histological analysis.

### Hematologic findings

Nodule formation and inflammation are ubiquitous within the spectrum of FD. This feature highlights the role that the hematopoietic system may play in the disease. The nodules are composed of foamy histiocytes and macrophages. This distinctive foamy phenotype is caused by the accumulation of storage material [98–101, 106, 107]. Ultrastructural analysis of nodules has revealed the presence of Zebra bodies and curved semi-linear tubular bodies (Farber bodies) [108–110]. Bloodwork samples from patients have also revealed an increased leukocyte count and erythrocyte sedimentation rate and moderately elevated plasma chitotriosidase and C-reactive protein (CRP) in severe cases [54, 111–115]. The formation of nodules and histiocytic infiltration may extend beyond the extremities and joints, and it has also been observed within the reticuloendothelial system, including the bone marrow, liver, lung, lymph node, and spleen, as well as the thymus and heart, in a number of patients [106, 116, 117]. In one case, solely the presence of invading histiocytes in a patient’s bone marrow aspirate led to the appropriate clinical identification of FD [118].

Several other hematologic findings have been reported. Enlarged lymph nodes have been noted in autopsy reports [2, 77, 116, 119]. Lymphadenopathy and calcification of the axillary lymph nodes have been detected on X-rays [100]. Finally, anemia, thrombocytopenia, and the presence of nucleated red blood cells have also been reported in FD patients [99, 100, 116].

### Neurological findings

Neurological manifestations are usually only seen in patients with Type 5 or classical FD [8]; the epileptic picture that is characteristic of SMA-PME is described in a separate paragraph below. Neurological involvement in FD is broad and can affect the central or peripheral nervous systems. Within the brain, hydrocephalus and cortical brain atrophy have been detected by magnetic resonance imaging [120, 121]. Storage pathology has been reported in a variety of neural tissues, including the anterior horns of the spinal cord, the brain stem, the cerebral cortex, and the cerebellum [17, 120, 122–124]. Storage pathology has also been reported in cells of the peripheral nervous system (PNS), where both myelinating and non-myelinating Schwann cells have large membrane-bound inclusions [60, 124, 125]. Pathology descriptions suggest that compression of the axonal body may affect proper nerve conduction [98, 124, 125]. A number of case reports have documented the occurrence of seizures and developmental delay leading to intellectual disability [38, 120, 122, 124]. Due to the pathology in the anterior horn cells and peripheral neuropathy, patients may also present with hypotonia, muscle weakness, and atrophy, leading to them requiring wheelchairs [38, 120, 122–124].

### Pulmonary findings

Beyond the development of the cardinal phenotypes, pulmonary complications are one of the more common occurrences in both classic and attenuated variants of FD [9]. Clinical signs may include sternal retraction, expiratory stridor, aphonia, and labored breathing [1, 38, 77, 97, 102]. As mentioned above, when nodule formation in the larynx and upper airway is extreme, tracheostomy may be required [56, 102, 126]. X-rays have shown presence of consolidation, nodular opacities, and lung atelectasis [86, 97, 102, 125]. Bronchial alveolar lavage and post-mortem analyses of patients have revealed significant inflammation with large lipid-laden macrophages and cellular infiltration throughout the bronchioles and alveoli [1, 67]. The lung tissue of one patient was described as poorly expanded with excessive connective tissue, and its ultrastructural analysis revealed lung histiocytes containing curvilinear storage bodies [123]. Pulmonary distress, infection, and pneumonia are the main causes of mortality [2, 8, 97, 101, 123, 127].

### Ophthalmic findings

Ocular manifestations have mostly been associated with the classic form of FD and those with neurological involvement [8]. In Farber’s original description of the disorder, he reported that his second patient was blind; however, limited analysis was performed [2]. A variety of ophthalmic findings have been documented in the literature; the most common sign is a cherry red spot [77, 115, 125, 126, 128, 129]. Additional ocular manifestations include retinal opacification, corneal opacities, and macular degeneration [10, 59, 128, 130]. Other findings related to the eyes have included the presence of xanthoma-like growths in the conjunctiva, poor visual fixation, and nystagmus [102, 120, 127]. Post-mortem analyses of the eyes showed no abnormalities in the anterior segment, but the posterior segment contained birefringent lipids within the ganglion cell layer and displayed significant storage pathology in other cell types in the eye [128, 131].

### Gastrointestinal findings

There are several cases in the literature describing gastrointestinal manifestations of FD. Persistent diarrhea has occasionally been seen in infants [99, 110]. One patient also exhibited extensive gastrointestinal lesions with widespread erosion of the gastrointestinal mucosa [110]. Another study that biopsied colonic tissue in a patient with severe disease demonstrated an increased level of apoptosis of cells within the crypt of the colon. This study also demonstrated that the caspase-3 positive cells co-localized with cells that were positive for GD3 gangliosides, concluding that colonocyte apoptosis may be triggered by the synthesis of GD3 as a consequence of ceramide accumulation [132].

### Hepatic findings

A palpable liver and hepatomegaly are commonly reported in patients with the classic variant of FD [1, 59, 67, 86, 96]. Zebra bodies and Farber bodies have been observed in hepatocytes, endothelial cells, and Kupffer cells [133, 134]. The most significant liver pathology seen is in patients with severe type 4 FD [8]. Infants have presented with cholestatic jaundice, ascites, liver fibrosis, and elevated liver enzymes [11, 56, 135]. In a unique case, a 6-month-old infant showed significant liver failure and was misdiagnosed with neonatal hepatitis; he/she underwent liver transplantation, which subsequently normalized the liver function [56]. FD was properly diagnosed in that instance after the appearance of nodules and histiocytic infiltrates. In these few severe cases, the enlargement of visceral organs and histiocyte formation may mask or precede the appearance of nodules [56].

### Bone findings

When joint involvement is present in FD patients, there may also be juxta-articular bone erosion and demineralization [86, 87, 96, 103]. In addition to the joints, bone erosion has been observed in long bones, metacarpals, metatarsals, and phalanges [111, 116, 136–138]. Osteoporosis is often progressive during the course of disease [97, 99, 102]. One patient, a 9-year-old girl, grew a tumorous osseous lesion in her spine, resulting in destruction of the odontoid by inflammatory cells. She underwent two HSCTs, which improved her mobility, but episodes of myoclonic epilepsy were still persistent [139]. In the milder spectrum, Bonafé et al. presented a case series of three siblings who displayed peripheral osteolysis between the ages of 40–60 years [93]. The patients all had shortened fingers and toes, as well as redundant skin. One of the siblings had limited movement of his knees and toes [73]. An unrelated 29-year-old patient also displayed deformities of the hands, demonstrating shortened fingers and redundant skin [97]. These patients had longer than average lifespans and were not formally diagnosed with FD until well into adulthood, which indicates that such milder cases may be underrepresented.

### Dermatological findings

In addition to the formation of subcutaneous nodules, skin lesions and plaques have been reported in some FD patients [99, 140, 141]. Analyses of dermal biopsies have revealed hyalinized collagen in the dermis, hyperkeratosis, and the presence of large foamy histiocytes [99, 131, 134]. The storage pathology in dermal tissue and histiocytes revealed the presence of Farber bodies [134, 141, 142]. A rare presentation featured an infant with clinical signs that overlapped with stiff skin syndrome [113]. The infant displayed thick indurated skin since birth, a stiff neck, and scleroderma-like areas; he/she died at around 2 years of age [113]. Recently a study has demonstrated that heterozygous *ASAH1* mutations may increase the susceptibility for keloid formation. This report performed genetic analyses on a Yoruba family in Nigeria, and of 24 members, 9 had keloids and 2 others had hypertrophic or stretched scars [52]. The L386P mutation (clinVar ID SCV000538196) was identified through a combination of linkage analyses and exome sequencing [52]. The appearance of keloids in this family ranged from 2 to 57 years of age. Additionally, the locations of keloid formation varied. Unfortunately, no lipid analysis or enzyme activity was reported. However, this variant nonetheless expands the clinical picture of ACDase deficiency [52].

### Hydrops Fetalis

In the literature to date, there have been two FD patients presenting with hydrops fetalis [34]. One report is of a 29-week-old stillborn fetus with mild internal hydrops, a well-preserved spleen, and the presence of foamy cells [117]. The second report is of a 3-day-old neonate with an extreme phenotype of hydrops [106, 143]. The latter infant presented with an enlarged abdomen filled with hemorrhagic ascites, hepatosplenomegaly, and many white nodules on the peritoneal surfaces of the liver, spleen, and other organs. These two cases of fetal hydrops represent the shortest-lived patients recorded in the Farber literature.

### Spinal muscular atrophy with progressive myoclonic epilepsy (SMA-PME)

A new variant of ACDase deficiency has emerged that shares no classical signs and symptoms of FD. These patients have a separate disease called spinal muscular atrophy with progressive myoclonic epilepsy (SMA-PME) (OMIM #159950). SMA-PME was first described in 1978 by Jankovic and colleagues. He described patients from a family in Louisiana and Texas who first developed muscle weakness and wasting, which gradually progressed to jerking of the limbs and myoclonus [144]. Most patients who suffer from SMA typically have a mutation in *SMA1* or *SMD2* [36]. However, some patients who have SMA-PME have now been identified to carry mutations in *ASAH1* [36, 44, 46, 91, 145, 146]. To the best of our knowledge, there have been 23 confirmed cases of SMA-PME with *ASAH1* mutations reported in the literature to date (Table 1). Additionally, from 1978 to 2009, 20 cases were reported to have a SMA-PME-like clinical presentation, which include the original case described by Jankovic [144, 147–151].

Symptoms of SMA-PME may appear as early as 2 years of age [44] and include increasing difficulty in walking, sporadic falls, muscle weakness, and tremors [35, 36, 151]. Development of lower motor neuron disease in the form of muscle weakness is often the first manifestation of SMA-PME in patients [152]. Muscle weakness has been reported in young children between 3 and 7 years of age up to adolescents at 15 years of age [44, 145]. Lower motor neuron disease also affects the respiratory muscles. Death is usually attributed to respiratory failure and has been recorded as early as the teenage years [36, 44, 91, 145]. Epilepsy usually develops after the onset of neuronal disease during late childhood, though exceptions have occurred such as in the case report by Filosto and colleagues where two sister-patients both developed an adult SMA phenotype with no myoclonic epilepsy [35, 47]. The most common form of epilepsy is myoclonic seizures which appear as a series of shock-like upper limb proximal jerks [152]. Action myoclonus and myoclonic status have also been documented in some patients [44]. Lastly, other manifestations include development of generalized tremors, scoliosis, and sensorineural hearing loss [44, 91, 145, 152]. As the disease progresses, patients experience increasing seizure activity [151]. Impaired mobility, cognitive decline, and difficulty swallowing occur near the end of life [152].

Generalizations of the clinical picture should be made with caution since the number of identified SMA-PME patients is limited, and most reported cases share the same T42M mutation (Table 4). However, several cases encourage a broader understanding of SMA-PME. For example, the first description of an adult SMA patient with a mutation in *ASAH1* did not present myoclonic epilepsy [47]. Another patient presented with eyelid myoclonic status epilepticus, in addition to muscle weakness, which has not been previously observed in SMA-PME [153].

### Phenotypic variability in ACDase deficiency

*ASAH1* mutations seem to result in two separate disorders, demonstrating the broad importance of ACDase for the proper maintenance of health. We have highlighted the diverse clinical spectrum that can be seen in various forms of ACDase deficiency. Interestingly, phenotypic variability is also seen in reports involving siblings. In one such case, one sibling demonstrated a classic Farber phenotype and died at 6 months of age, whereas the other sibling survived to 12 weeks of age and had extreme histiocytic infiltration throughout the body [67]. Surprisingly, post-mortem analyses of liver tissue from both patients revealed a similar level of enzyme activity [67]. Another case showed hepatosplenomegaly in a 3-month-old male [119]. While no nodules were noted in this patient, histiocytosis was the dominant phenotype. His sister, who was 5 and a half months old, displayed a classical phenotype of FD [119]. Fiumara et al. featured two sisters and one female cousin with a mild variant of FD and significant symptom variability [97]. Clinically, all three patients displayed nodule formation, joint involvement, and the presence of erosions [97]. However, variability in symptom onset and longevity was observed. One sister developed symptoms in her second year of life and lived to 30 years of age, whereas the other sister was symptomatic at 20 months of age and died when she was 18 years old. While ACDase enzyme activity was not reported for the shorter-lived sister, the assay was performed on cells cultured from the cousin who developed symptoms even earlier and died the earliest (at 11 years of age), the long-lived sister, and an established FD control cell line (FD patient who died at 1.8 years of age). In this enzyme activity assay, both the long-lived sister, the cousin, and the FD control showed an enzyme activity between 4 and 6% of normal controls [97]. Presumably, the three mild FD patients shared a similar mutation, yet there was obvious variability in symptom onset and patient longevity [97]. Similarly, enzyme activity for classical patients, who have shorter lifespans, may be comparable to those of patients who are long-lived. Therefore, while enzyme activity is important for diagnosis of FD, there is not a complete correlation between in vitro enzyme activity levels and patient outcomes.

## Research, treatment and future therapy

### Animal models

An ACDase knock-out mouse model was previously generated through insertional mutagenesis into the *Asah1* gene. Heterozygous mice (*Asah1*^*+/−*^) did not show any overt changes in phenotype and had a normal life span of at least 1.5 years [154]. However, analyses of the organs of heterozygous mice 6 months of age and older revealed lipid accumulation and inclusions in the liver, lung, skin, and bone [154]. The heterozygous liver was the most affected; it became fibrous and pale. While most hepatic cell types were filled with lipids, the most significant effect was observed in Kupffer cells. By 9 months of age, some ceramides were also elevated in the heterozygous animals, in which the greatest accumulation was detected in the liver, with a 1.5–2-fold increase compared with wild-type animals. Homozygous mice (*Asah1*^−/−^) were embryonic lethal; none were detected at day E8.5 or later [154]. A second knock-out mouse was generated via a targeted ES cell clone [155]. Analyses of this model demonstrated that homozygous embryos did not survive beyond the 2-cell to the 4-cell stage and underwent apoptotic cell death, highlighting the importance of ACDase as a vital enzyme for early embryonic development.

A tamoxifen-induced conditional *Asah1* knock-out mouse has also been developed [156]. Intraperitoneal delivery of tamoxifen in 5-week-old female mice resulted in impaired fertility due to lack of mature follicles in the ovaries. The follicles were not able to fully develop, and apoptosis occurred between the transition from the secondary to the antral stage. This observation supports the essential role of ACDase in ovary maturation and its importance in fertility [156]. Tamoxifen injection showed variable penetrance, where 100% *Asah1* ablation was reported in skin and 70% in ovaries [156]. Reports using this conditional knockout have focused exclusively on the ovary phenotype. While classical and severe cases of FD do not survive to sexual maturity, data derived from these studies may prove relevant with regard to mild and attenuated FD patients. While no overt FD phenotypes have been reported, this model nonetheless may serve as an important tool for fertility studies.

Finally, a knock-in model has also been developed, in which an *ASAH1* patient mutation (P362R) was introduced into the analogous murine locus (P361R), resulting in a mouse that recapitulates many of the phenotypes observed in classical cases of FD [157]. The P362R mutation has been identified in two patients with FD. One patient, who died at 1.5 years of age, had a classical form of FD and was homoallelic for the mutation [41]. The other patient, who died at 8 years of age, was heteroallelic for P362R and E138V [41]. Furthermore, this mutation site was selected because it represents the most conserved region of the gene between the species [157]. Homozygous (*Asah1*^P361R/P361R^) mice have a decreased lifespan and reduced weight. These mice develop a significant inflammatory phenotype and the accumulation of large foamy macrophages in many tissues. Recent studies have also shown that these mice have impaired hematopoiesis, central nervous pathology, abnormal skin development, and impaired lungs [94, 158–160]. The *Asah1*^P361R/P361R^ model does not develop nodules, but it does exhibit many features that are seen in patients, such as inflammation; enlarged organs, including hepatosplenomegaly; respiratory distress; and neurological and behavioral impairment [94, 158–160]. Animal studies have thus provided key insights into ACDase biology and *Asah1* mutant pathology. In addition, they have and will continue to serve as important models that will ultimately guide and inform the use of future therapies in patients.

### Current treatment

There is currently no cure for ACDase deficiency. Current treatment strategies focus on symptom management. Anti-inflammatory medications and physical therapy can help address pain and mobility issues [18, 161, 162]. Surgical intervention may occasionally be applied for the removal of nodules in the hands and oral cavity [138, 163]. In one severe case in which a patient was misdiagnosed with hemangioendothelioma, a series of five surgeries to remove sacrococcygeal masses and three surgeries for scalp masses were performed over the course of a patient’s life. He/she eventually expired at 5 years of age [164]. HSCT is another therapeutic option and has been demonstrated to substantially improve mobility and pain in a number of FD patients lacking CNS involvement [165, 166]. Early studies in which HSCT was performed in two patients with classical FD with CNS complications were promising because they showed an elevation in ACDase activity and resolution of voice hoarseness, subcutaneous nodules, and painful joints [167, 168]. However, in both cases, HSCT did not reverse the neurological phenotypes, and the patients deteriorated over time. A recent article has provided long-term follow-up data on 10 FD patients who underwent HSCT within the last 15 years [169]. Eight of the 10 transplanted FD patients in that study are still alive with a mean survival time to date of 10.4 years [169]. Inflammatory joint disease was resolved in all the surviving patients, respiratory findings were variable, and the neurological deficits persisted (and even progressed in some of the patients) [169]. Despite the scarcity of patient data, HSCT appears to be a promising treatment for mild and attenuated FD.

For SMA-PME, most patients are prescribed anti-epileptic drugs to assist with seizure control, with mixed efficacy [146, 153]. Since respiratory complications are progressive, some patients may also require mechanical ventilation and gastric feeding [44, 153].

### Gene therapy

ACDase deficiency is an attractive target for gene therapy because it is caused by a single gene defect. In fact, several gene therapies for monogenic lysosomal storage disorders are currently being investigated in clinical trials [170–172]. In the context of ACDase deficiency, one early study demonstrated that FD patient cells recovered ACDase activity when the cells were infected with an onco-retroviral vector that engineered expression of human ACDase [173]. This study confirmed that the transduced cells had increased ACDase activity and normalized ceramide levels [173]. Additionally, the treated cells could also cross-correct untreated cells when supplemented with medium from transduced cells that secreted human ACDase [173]. Through the mannose-6-phosphate receptor pathway, the non-infected cells acquired functional enzyme, demonstrating the effect of metabolic co-cooperativity. A later study reproduced this same effect using lentiviral vectors as the delivery vehicle and showed successful gene correction in hematopoietic stem cells [174]. That same study also showed that direct injection of vector into murine neonates could provide long-term expression of ACDase for up to 13 weeks [174]. This same approach was applied to the P361R FD mouse model and demonstrated an increased lifespan from 9 to 10 weeks to 16.5 weeks of age [157].

Ex vivo gene therapy is a treatment strategy that may deliver a longer-lasting therapeutic benefit than traditional HSCT. In this approach, stem, progenitor, or differentiated cells are isolated from a patient or donor, modified by genetic correction, and subsequently transplanted back into the patient [175, 176]. HSCs are a promising cell type for such gene therapy strategies since they are readily accessible and easily separated from a patient’s blood and can expand/differentiate into long-lived cell types [177, 178]. Ex vivo gene therapy followed by transplant represents an improvement over HSCT alone because the transduced cells express enzyme derived from the therapeutic vector in addition to their endogenous gene expression, which, in theory, allows for increased enzyme production, lysosomal activity, and potential cross-correction.

Many active gene therapy protocols are investigating such ex vivo HSCT transductions/transplantations to treat genetic disorders [179]. Ex vivo gene therapy followed by transplant may circumvent the limitations of HSCT alone to improve neurological symptoms, as in the case of metachromatic leukodystrophy [180]. In the case of ACDase deficiency, ex vivo transduction followed by HSCT is also a promising option. A series of proof-of-concept studies have demonstrated the successful transduction of the huACDase cDNA into murine CD34+ stem/progenitor cells and later into analogous cells from non-human primates [174, 181]. In the latter study, higher than normal ACDase enzyme activity was detectable in peripheral blood cells, in the bone marrow, the spleen and liver for more than a year [181]. Additionally, the animals had decreased ceramide levels [181].

At the time that this manuscript was written, a gene therapy trial was initiated for the treatment of SMA type I (clinicaltrials.gov ID NCT02122952). This trial involves the use of adeno-associated virus serotype 9 (AAV9), a non-integrating virus that encodes the SMA1 cDNA, infused through a peripheral vein. While the trial is still ongoing, preliminary data demonstrate a reduced need for pulmonary support, and patients could feed themselves, indicating a potential improvement in swallowing function [182, 183]. Although these results are for a different type of SMA, it is possible that a similar gene therapy approach may also be promising for patients with the SMA-PME phenotypes.

### Enzyme replacement therapy

Enzyme replacement therapy (ERT) is currently the standard of care for several LSDs. Since early studies demonstrating the efficacy of ERT in Gaucher disease, this treatment strategy has been developed for a wide assortment of LSDs. It has been implemented to treat Pompe disease, Fabry disease, MPSI, II, VI, neuronal ceroid lipofuscinosis type 2 (CLN2), and Niemann-Pick B [184–190]. ERT with rhACDase is currently under development and represents a promising therapy for ACDase deficiency and several other conditions in which ceramide accumulation is pathologic, such as cystic fibrosis [191, 192]. Currently, large volume production of rhACDase is achieved by the amplification and transfection of Chinese hamster ovary (CHO) cells [193]. Overexpression of ACDase in CHO cells results in the secretion of enzyme into the medium, which is then purified by a series of chromatography steps [193].

A recent proof-of-concept study using the CHO-derived rhACDase as treatment in the P361R FD mouse model has shown promise [193]. Treatment with the recombinant enzyme resulted in decreased ceramide accumulation, less macrophage infiltration, lower MCP-1 expression, and a normalized spleen weight in FD mice [193]. This initial study holds promise for future FD treatments, but further investigations are required to better delineate the dose response in this model and to determine how this effect can be better translated to the human variant of FD or SMA-PME. One limitation of ERT is a reduced ability to cross the blood-brain barrier, which represents an issue for those LSDs that manifest with neurologic components, such as severe cases of FD. However, targeted CNS administration of enzyme has been observed to circumvent this limitation, and the use of fusion proteins with CNS-targeting moieties is currently being evaluated as a promising method for enzyme delivery to the CNS [194].

## Conclusion

Over 70 years have passed since Farber’s Mayo Foundation lecture. Included in this historic transition is a brief transcript where Farber states: *“The clinical picture I describe may be found to be typical for these 3 cases and may not be encountered in the next 20 or 30. We should, with a disease of this kind, expect to see a number of unrelated clinical pictures in the future*” [1]. Farber’s comment and insight are highly relevant to this day. ACDase deficiency is a spectrum disorder that includes FD, SMA-PME, and potentially keloid formation or susceptibility to schizophrenia. Even amongst the individual conditions, there is a wide clinical spectrum. In mild cases, a misdiagnosis or a delay in diagnosis could impact the treatment plan and adversely affect the ability to properly manage symptoms [34]. A natural history study is currently underway on clinicaltrials.gov (ID NCT03233841), which aims to gain greater insight into the natural history of ACDase deficiency through retrospective and prospective patient data. It also aims to establish clinical information, biomarkers and other functional data to access the efficacy of future therapies, such as rhACDase ERT. The establishment of a complete natural history will greatly improve and potentially fill in gaps in the current definition of ACDase deficiency. Finally, due to the wide spectrum of clinical presentations, the precise number of patients is likely to be underrepresented. An improved understanding of the disease and increasingly effective knowledge translation will allow more patients to be identified, efficiently diagnosed, and effectively managed.

## Additional file

Additional file 1: A description of the literature search method used to identify FD and SMA-PME patients from research articles and case reports. (DOCX 20 kb)

## Abbreviations

AAV9: Adeno-associated virus serotype 9
ACDase: Acid ceramidase
CHO: Chinese hamster ovary
CLN2: Neuronal ceroid lipofuscinosis type 2
CRP: C-reactive protein
CNS: Central nervous system
EEG: Electroencephalogram
EMG: Electromyogram
ERT: Enzyme replacement therapy
ESI/MS: Electrospray ionization mass spectrometry
FD: Farber disease
JIA: Juvenile idiopathic arthritis
LSD: Lysosomal storage disorder
MCP-1: Monocyte chemoattractant protein 1
MS: Mass spectrometry
PNS: Peripheral nervous system
rhACDase: Recombinant human ACDase
SAPs: Sphingolipid activator proteins
SMA-PME: Spinal muscular atrophy with progressive myoclonic epilepsy
