# Supplementary material for: Acid ceramidase deficiency: Farber disease and SMA-PME
Source: Orphanet J Rare Dis. 2018 Jul 20;13:121. doi: 10.1186/s13023-018-0845-z (PMC6053731; doi:10.1186/s13023-018-0845-z)
Supplement: Supplementary file 1 — A description of the literature search method used to identify FD and SMA-PME patients from research articles and case reports. (DOCX 20 kb) [file 13023_2018_845_MOESM1_ESM.docx]

## Additional file 1

Literature Search Method

A literature search was performed in PubMed and Google Scholar using the following search terms in varying combinations: Farber disease, Farber lipogranulomatosis, acid ceramidase deficiency, ceramide, *ASAH1*, acid ceramidase, SMA-PME, spinal muscular atrophy progressive myoclonic epilepsy, case report. The search was limited to publications generated between 1952 and 2017. A total of 203 articles was found. We narrowed our search to articles that contained patient information, resulting in 124 articles. Analysis of these articles resulted in the identification of 152 patients with FD and 43 with SMA or SMA-like phenotypes. We separated the patients by variant and severity. A custom classification system was created as follows. Classic & Severe FD was considered if at least 3 of the following occurred: 1) death by 4 years of age, 2) onset of disease by 6 months, and 3) the presence of cardinal phenotypes and hepatosplenomegaly or neurological phenotypes. Additionally, if the case stated a severe or classic case, the patient was automatically placed in that category. The Mild & Intermediate category was considered if at least 2 of the following observed: 1) lived past 6 years of age, 2) onset of disease occurred after 1 year of age, and 3) no neurological or hepatosplenomegaly phenotypes. Additionally, if the case was stated a mild, intermediate, attenuated or long-lived, the patient was automatically placed in that category. Unspecified cases included an article that mentioned patients who were diagnosed with FD but included insufficient data to determine the variant type. The SMA-PME group included all those patients who had a mutation in *ASAH1* and were described as SMA-PME patients. The SMA-PME-like group included those diagnosed prior to the genetic connection established by Zhou and colleagues in 2012. In this case, a patient was considered to have SMA-PME if they developed muscle weakness, had a history of seizures and were not placed in a separate SMA subgroup. For all cases, we included the age of symptom onset, age of death and last documented age if death was not included. Only cases with the indicated information were included in the average age calculations.
